# Supplementary material for: Complexome profiling of the Chlamydomonas psb28 mutant reveals TEF5 as an early PSII assembly factor
Source: Plant Cell. 2025 Mar 17;37(6):koaf055. doi: 10.1093/plcell/koaf055 (PMC12164590; doi:10.1093/plcell/koaf055)
Supplement: koaf055_Supplementary_Data [file koaf055_supplementary_data.zip › psb28_interactive.zip!/plots/intensity/qualityPlots/peptides/CGLD1_Cre02.g084350.t1.2.html]

### Cre02.g084350.t1.2

Trivial name: CGLD1  
SeqCoverage [median]: 13.00  
Mass: 25.20 kDa  
  
 

| Sequence | MissedCleavage | Mass [Da] | wtIntensity | muIntensity | Score |
| --- | --- | --- | --- | --- | --- |
| DAGESAAEEKEEAQEVVDGLK | 1 | 2203.01 | 15377.55 | 63589.30 | 218.69 |
| DAKDAGESAAEEKEEAQEVVDGLK | 2 | 2517.17 | 486.48 | 918.39 | 44.63 |
| EEAQEVVDGLK | 0 | 1215.60 | 0.00 | 1008.06 | 90.15 |
| SSSEDALK | 0 | 835.39 | 0.00 | 332.45 | 75.21 |
|  |  |  | 15864.0 | 65848.2 |  |
